# Emerging targets of α-synuclein spreading in α-synucleinopathies: a review of mechanistic pathways and interventions

**DOI:** 10.1186/s13024-025-00797-1

**Published:** 2025-01-23

**Authors:** Grace Kuo, Ramhari Kumbhar, William Blair, Valina L. Dawson, Ted M. Dawson, Xiaobo Mao

**Affiliations:** 1 Neuroregeneration and Stem Cell Programs, Institute for Cell Engineering, Johns Hopkins University School of Medicine, Baltimore, MD 21205 USA; 2 Department of Neurology, Johns Hopkins University School of Medicine, Baltimore, MD 21205 USA; 3 Adrienne Helis Malvin Medical Research Foundation, New Orleans, LA 70130-2685 USA; 4 Department of Physiology, Johns Hopkins University School of Medicine, Baltimore, MD 21205 USA; 5 Solomon H. Snyder Department of Neuroscience, Johns Hopkins University School of Medicine, Baltimore, MD 21205 USA; 6 Department of Pharmacology and Molecular Sciences, Johns Hopkins University School of Medicine, Baltimore, MD 21205 USA; 7 Institute for NanoBioTechnology, Johns Hopkins University, Baltimore, MD USA; 8 Department of Materials Science and Engineering, Johns Hopkins University, Baltimore, MD USA

**Keywords:** Prion-like, α-synuclein, Spreading, Receptor, Therapeutic targets

## Abstract

α-Synucleinopathies constitute a spectrum of neurodegenerative disorders, including Parkinson’s disease (PD), Lewy body dementia (LBD), Multiple System Atrophy (MSA), and Alzheimer’s disease concurrent with LBD (AD-LBD). These disorders are unified by a pathological hallmark: aberrant misfolding and accumulation of α-synuclein (α-syn). This review delves into the pivotal role of α-syn, the key agent in α-synucleinopathy pathophysiology, and provides a survey of potential therapeutics that target cell-to-cell spread of pathologic α-syn. Recognizing the intricate complexity and multifactorial etiology of α-synucleinopathy, the review illuminates the potential of various membrane receptors, proteins, intercellular spreading pathways, and pathological agents for therapeutic interventions. While significant progress has been made in understanding α-synucleinopathy, the pursuit of efficacious treatments remains challenging. Several strategies involving decreasing α-syn production and aggregation, increasing α-syn degradation, lowering extracellular α-syn, and inhibiting cellular uptake of α-syn are presented. The paper underscores the necessity of meticulous and comprehensive investigations to advance our knowledge of α-synucleinopathy pathology and ultimately develop innovative therapeutic strategies for α-synucleinopathies.

**Graphical Abstract:**

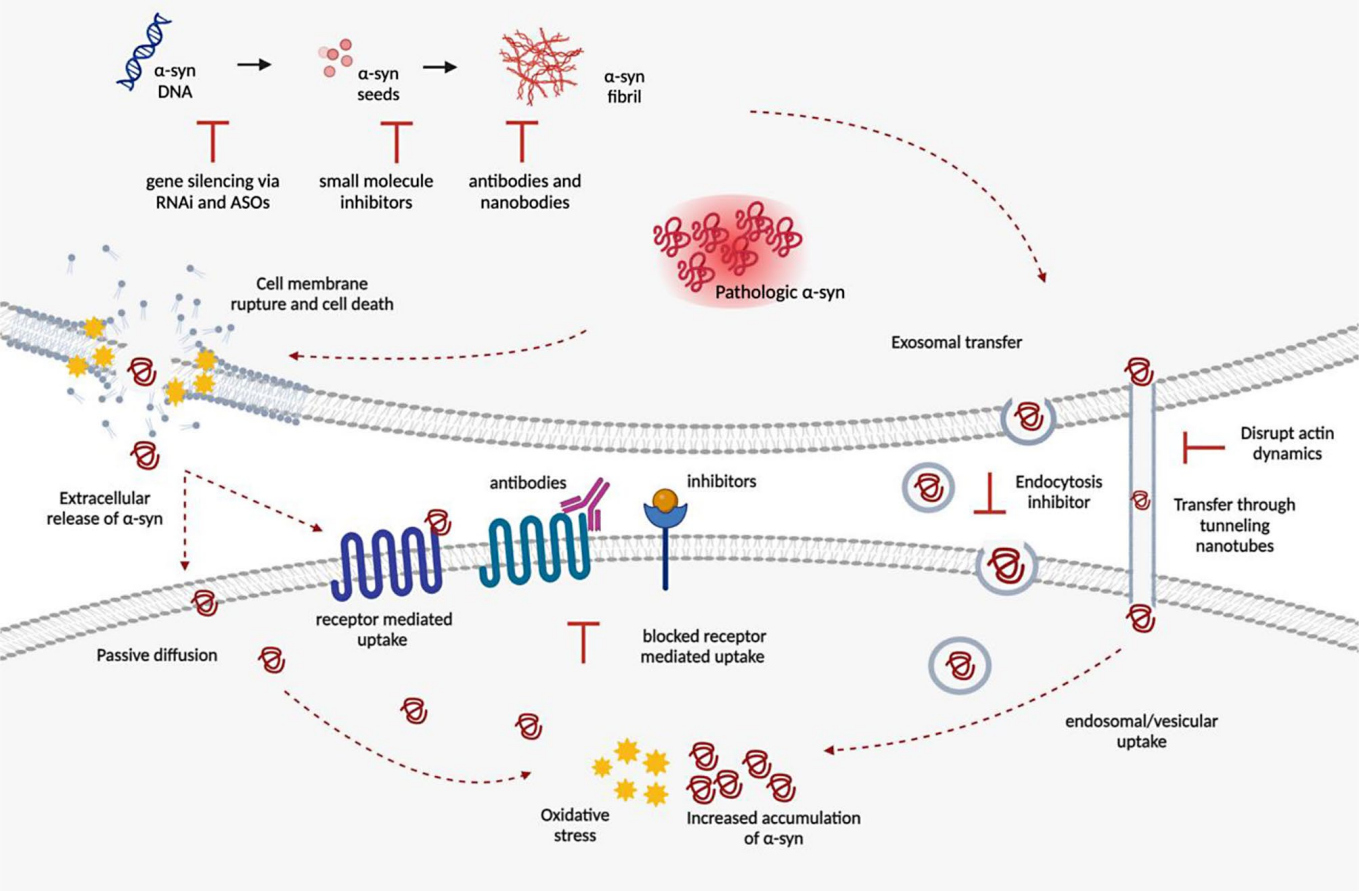

## Background

The significance of α-synuclein (α-syn) in the pathogenesis of Parkinson’s disease (PD) was proposed more than twenty years ago through the discovery of genetic forms of PD. Subsequent studies have demonstrated that abnormal aggregates of α-syn, including Lewy bodies (LB), Lewy neurites, and glial cell inclusions, are central to a diverse range of neurodegenerative conditions collectively termed as α-synucleinopathies. These diseases include idiopathic PD, Dementia with Lewy bodies (DLB), Multiple systems atrophy (MSA), Pure autonomic failure (PAF), and rapid eye movement (REM) sleep behavior disorder (RBD) [1]. 

α-Syn is comprised of 140 amino acids, split into 3 main regions responsible for different biological and molecular functions [2]. While native α-syn has an ‘unfolded monomer’ conformation, it can naturally exist as a folded tetramer with a minimal propensity for amyloid-like aggregation [1]. In its physiological state, α-syn is thought to modulate various cellular processes, including synaptic function and neurotransmitter release, playing a pivotal role in maintaining synaptic plasticity [2]. Despite these known roles, the precise functions of α-syn remain largely elusive.

α-Syn pathology is initiated by the intramolecular unfolding of α-syn within and through the secondary nucleation process. This leads to the rapid multiplication of α-syn fibrils, aggregation, and neurotoxicity [3]. Specifically, α-syn in its monomeric form interacts with the C-terminal ends of α-syn fibrils through its own N-terminal ends. During this phenomenon, molecular interactions trigger the unraveling of protein structures, revealing sections prone to clustering [1], specifically the C terminus which further facilitates receptor binding [4]. Once this aggregation catalyzes a new generation of aggregates, the lipophilic nature of the oligomers formed can lead to cell membrane permeability. In addition, a rapid increase of basal cytosolic Ca^2+^ levels occurs, which is compounded by the limited abilities of neurons to achieve homeostasis, ultimately leading to disease progression [1]. Exocytosis releases fibrils into the extracellular space, where they may be internalized by neighboring cells via endocytosis; even when these exogenous pathological fibrils are truncated and lose their C termini through degradation at new sites, they remain capable, perhaps even more so, of seeding the aggregation of endogenous α-syn, perpetuating the pathological process [5]. Through this mechanism, research has purified α-syn seeds that can be used to model aggregated forms of amyloid-like fibrils, otherwise known as pre-formed fibrils (PFFs) [4]. Both in vitro and in vivo, misfolded α-syn propagates in a prion-like manner. Minimal quantities of PFFs are required to trigger the conversion of native α-syn to its toxic aggregated form. Consequently, α-syn fibrils spread and affect healthy brain areas, leading to neural degeneration and the advancement of various α-synucleinopathies [5]. 

Within the therapeutic landscape for neurodegenerative diseases, targeting α-syn pathology is a promising strategy. A fundamental aspect of this approach involves a nuanced understanding of receptors that mediate cellular uptake, mechanisms that contribute to intercellular spread, as well as the pathological agents that contribute to α-synucleinopathy progression. Currently, there are no known disease-modifying treatments available for α-synucleinopathies. Thus, this review seeks to focus on membrane receptors and proteins that facilitate α-syn propagation, which constitute viable intervention points to modulate disease progression. Additionally, addressing intercellular spreading pathways can further contribute to derived therapeutics intervening at different times. A comprehensive approach to this problem must also consider the pathological aggregates of α-syn as these aggregates are the primary cause of cytotoxicity and neuronal cell death. By aiming at these three main targets, the review aims to provide current assessments of therapeutics for α-syn pathology and highlight potential areas for further investigation.

## Targeting membrane receptors and proteins

Targeting membrane receptors presents a potentially important therapeutic avenue for influencing the course of α-synucleinopathies due to their role in mediating the cell-to-cell transmission of α-syn aggregates [5]. These receptors are integral to the uptake and propagation of pathological α-syn species across neural networks. By modulating these receptor-mediated pathways, it is possible to interrupt the prion-like spread of α-syn. Specifically, membrane receptors illustrated in Table 1 are involved in either the binding or internalization of α-syn aggregates, a process that not only contributes to the spread of pathology but also triggers downstream neuroinflammatory and neurodegenerative processes. The binding of α-syn to these receptors can activate intracellular signaling pathways that exacerbate cell stress and neurotoxicity [1]. Therefore, the development of therapeutic agents that can inhibit these interactions or modify receptor activity could provide neuroprotection and slow disease progression.

### Membrane receptors and proteins

#### Low-density lipoprotein receptor-related protein 1 (LRP1)

LRP1, a critical member of the low-density lipoprotein receptor (LDL) receptor family, plays an essential role in the clearance of a range of ligands, encompassing apolipoprotein E (APOE) and amyloid-β (Aβ), in addition to their complexes [6]. LRP1’s expansive role in α-syn spreading has been endorsed by transgenic mouse models that feature neuronal deletion of the Lrp1 gene [6]. Research utilizing α-syn with modified lysine residues at the N-terminus further confirms that these lysine residues and the N-terminus are vital for α-syn’s interaction and uptake into cells [7]. Using both LRP1 knockout human induced pluripotent stem cell (iPSC) derived neurons and neuronal LRP1 deletion mice, a study demonstrated a significantly decreased amount of monomeric and oligomeric α-syn uptake while α-syn PFFs were mildly inhibited [6]. Such results suggest LRP1 only mediates the uptake of some forms of α-syn, specifically soluble α-syn. One possible explanation for this is that during the formation of α-syn PFFs, the N-terminus of α-syn may be less accessible, which could result in a limited interaction between PFFs and LRP1 [6]. Moreover, LRP1’s predilection for specific forms of α-syn, particularly soluble α-syn, and its unknown role in post-translational modifications of α-syn highlights the need for more nuanced exploration into the mechanistic pathways of α-synucleinopathies. Recently, it was shown that elevated levels of the intracellular domain LRP1 (LRP1-ICD) in brain microvascular endothelium were associated with increased uptake of α-syn PFFs, exacerbating endothelial damage and neuronal apoptosis. Overexpression of LRP1-ICD in brain capillaries intensified α-syn PFFs-induced vascular damage, pathology, and neuron death, leading to significant motor and cognitive impairment [8]. Collectively, these studies underscore LRP1 as a potential therapeutic target.

#### Lymphocyte-activation gene 3 (LAG3)

LAG3, a cell surface protein that belongs to the immunoglobulin superfamily, was shown to be an α-syn PFFs-binding protein. Investigations suggest that LAG3 facilitates the uptake of fibrillar forms of exogenous α-syn through endocytosis [9]. As an immune receptor, LAG3 is expressed on CD4^+^ and CD8^+^ T cells, and T_regs_ [10]. Recently, expression of LAG3 was shown to be present in oligodendrocytes, neurons, microglia, and endothelial cells [11–14]. Debate exists on LAG3’s neuronal expression where one study failed to detect LAG3 in neurons. This may be due to the lack of knockout controls and the use of T cells as a positive control. T cells highly express LAG3, potentially obscuring neuronal signals [15]. Public repositories including single-cell repositories indicate that LAG3 messenger RNA (mRNA) is expressed in neurons of various species, albeit at low levels [16–22]. Additionally, LAG3 reporter mice with a yellow fluorescent protein (YFP) knocked into LAG3 confirms unequivocally that LAG is expressed in neurons [23]. Furthermore, its colocalization with NeuN and its presence in wildtype (WT) but not LAG3 knockout mouse brain lysates support its role in α-syn pathology spread [23]. Studies have shown that either eliminating LAG3 expression or using neutralizing antibodies reduces the spread of disease caused by the cell-to-cell transmission of aggregated α-syn [9, 23]. Research in vivo has shown that knocking out LAG3 in an α-syn A53T transgenic mouse model leads to an extended lifespan and reduced behavioral deficits [11]. Similarly, a later study revealed that LAG3 also specifically binds to the fibrillar form of tau, but not the monomer. Deletion or inhibition of LAG3 in primary cortical neurons significantly reduced the internalization of tau PFFs, inhibiting tau propagation and neuron-to-neuron transmission. Interestingly, the authors found that heparin can inhibit Tau PFFs binding to LAG3 and cellular tau internalization. Cell surface binding assays showed significantly less binding in LAG3-transfected cells treated with heparin-induced Tau (hep-Tau) PFFs [24, 25]. Both tau and α-syn are known to aggregate and form toxic oligomers that drive pathology. Their relation to propagating disease progression can be seen as tau interacts with α-syn, accelerating its aggregation and enhancing seeding activity, leading to mitochondrial dysfunction, synaptic impairment, and neurotoxicity in vitro [26]. LAG3’s preference to bind to α-syn fibrils is due to the acidic C terminus, especially residues 118 to 140, of α-syn. The C terminus is condensed for aggregated α-syn; and thus phosphorylation at serine 129 increases α-syn’s binding affinity to other receptors, including LAG3’s D1 domain [4]. Most recently, LAG3’s uptake of α-syn PFFs has been further implicated in endothelial cells, leading to poly(ADP-ribose)-driven cell death. LAG3 has been confirmed as a receptor for pathogenic α-syn by another group in endothelial cells, where depletion of endothelial LAG3 significantly reduced α-syn PFFs-induced cerebral microvascular dysfunction. Additionally, endothelial LAG3 depletion inhibited α-syn PFFs-induced pathology propagation in the brain and alleviated cognitive dysfunction [13]. Thus, LAG3 is an important membrane receptor that mediates the uptake of pathologic α-syn.

#### Amyloid precursor-like protein 1 (APLP1)

APLP1, another α-syn PFFs-binding protein, belongs to the amyloid precursor protein (APP) family. It plays a role in α-syn transmission and pathology by binding LAG3 and forming a LAG3-APLP1 complex [23]. Phosphorylation of α-syn at serine 129 specifically promotes strong electrostatic interactions between fibrils and the positive surface of its corresponding receptors, further facilitating α-syn binding to APLP1 specifically through its E1 domain [23]. Endocytosis of pathologic α-syn occurs, in part, through APLP1 as APLP1 knockout primary cortical neurons have reduced uptake of pathologic α-syn. Pathologic α-syn-induced increases in pS129 levels are also reduced in APLP1 knockouts [23]. Together in conjunction with LAG3, the LAG3-APLP1 complex accounts for 40% of the binding of α-syn PFFs to primary cortical neurons and 70% of pathologic α-syn endocytosis. Anti-LAG3 antibodies were shown to disrupt the interaction between LAG3 and APLP1 by blocking LAG3’s D3 domain and thereby substantially blocking the internalization of α-syn, providing a new mechanistic perspective into the cell-to-cell transmission of pathologic α-syn [23]. 

#### Toll-like receptor 4 (TLR4)

TLR4, found on microglia and astroglia, is another member of the family that modulates the innate immune response. In microglia, TLR4 ablation caused diminished pro-inflammatory responses upon α-syn treatment and decreased reaction oxygen species (ROS) production [27]. In astroglia, TLR ablation displayed a suppressed pro-inflammatory response to α-syn and decreased ROS production. The uptake of α-syn by astroglia is not dependent on TLR expression. The same study also discovered that C-terminally truncated form of α-syn appears to be the strongest inducer of glial cell phenotypes [27]. Similarly, TLR4 knockout astrocytes exposed to extracellular α-syn showed reduced mRNA levels of pro-inflammatory cytokines and inflammatory enzymes [28], while TLR4 antagonists reduced ROS and neuronal cell death in primary neuronal cultures exposed to α-syn [29]. In the same study, prolonged exposure to low levels of α-syn oligomers sensitized TLR4 responses in astrocytes and microglia, inducing pro-inflammatory cytokines like TNF-α [29]. In addition, TLR4 expression was highest in the substantia nigra [29], and TLR4-deficient mice were protected against dopaminergic neuron loss induced by MPTP intoxication [30]. In a more recent study from a prodromal PD model of TLR4 deficient mice, decreased CD68 and p62 expression in microglia indicated low autophagy-lysosomal activity in response to human α-syn PFFs [31]. Microglia clearance of α-syn aggregates through autophagy seems to be mediated by the TLR4-NF-κB signaling pathway [32]. These studies collectively highlight TLR4’s dual role in driving neuroinflammation through astroglia and microglia while also facilitating the clearance of α-syn aggregates via autophagy.

#### α3-Na^+^/K^+^-ATPase (α3-NKA)

α3-NKA, a membrane protein, participates in maintaining the electrochemical gradients across cell membranes, managing cell volume, modulating neuronal excitability, and supporting various other cellular functions [33]. Research indicates that there is a significant association between α3-NKA and the oligomeric and fibrillar forms of α-syn, with the fibrillar forms exhibiting the strongest interactions. α-Syn forms clusters that entrap α3-NKA within the neuronal membrane, a phenomenon known as co-clustering. This process triggers a redistribution of α3-NKA that reduces its ability to pump out Na^+^ from neurons. Consequently, the impaired gradient instigates detrimental effects on neuronal health, primarily affecting the neuronal refractory period [34]. 

#### Integrin CD11b

Integrin CD11b, also known as the α-chain of integrin αMβ_2_, is a transmembrane receptor that facilitates cell-matrix adhesion [35]. Its implications in α-synucleinopathies can be seen in α-syn-induced microglial activation [36]. α-Syn aggregates binding to CD11b activates the Rho signaling pathway, which results in microglial NADPH oxidase (NOX2) activation. The same study also demonstrated mice lacking CD11b exhibiting higher resistance to the activation of NOX2 induced by α-syn in comparison to WT mice [36]. Another mouse study showed CD11b knockout significantly reducing locus coeruleus noradrenergic (LC/NE) neurodegeneration. This reduction was accompanied by mitigated microglial activation and decreased gene expression of proinflammatory cytokines [35]. 

#### Fc gamma receptor IIB (FcγRIIB)

FcγRIIB is a cell surface receptor involved in mediating immune responses. FcγRIIB specifically binds to the Fc region of IgG antibodies, and its activation leads to the suppression of immune responses [37]. Studies suggest that FcγRIIB acts as a receptor for aggregated α-syn [38, 39]. Once α-syn is bound to FcγRIIB on microglia, activation of Src homology 2 domain-containing protein tyrosine phosphatase 1 (SHP-1), an enzyme that regulates signaling pathways, leads to inhibition of microglial phagocytosis [38]. FcγRIIB was also implicated as a regulator of α-syn fibrils cell-to-cell transmission by activating lipid raft-dependent endocytosis and binding specifically to fibrils. Downregulation of FcγRIIB lowered transmission of α-syn between cells and further inhibited Lewy body-like inclusion formation in vitro [39]. These results suggest that bindings of α-syn to FcγRIIB can not only disrupt the immune response of microglial cells by inhibiting their phagocytotic abilities but also are involved in cell-to-cell transmission.

#### Heparan sulfate proteoglycans (HSPGs)

HSPGs, located on the cell surface and within the extracellular matrix, execute diverse functions, contributing significantly to cell adhesion, cell signaling, and extracellular matrix organization. Notably, these cell surface molecules interact with various ligands and play crucial roles in cellular processes. HSPGs, transactivators of transcription (TAT) peptide, are mainly implicated in the cellular endocytosis of monomeric tau. Specifically, genetic knockouts of a key enzyme in HSPG synthesis demonstrated a reduced level of tau uptake [40]. This process can happen in two distinct pathways; for large tau aggregates, a process known as macropinocytosis is used for internalization, while for tau soluble oligomers and monomers, receptor-mediated endocytosis is employed to cross the cell membrane [41]. Additional studies discovered that syndecan-related HSPGs are involved in the clathrin-independent internalization of tau fibrils through a mechanism associated with lipid rafts, consistent with the macropinocytosis pathway for tau fibril absorption [42]. Research using cell lines from the central nervous system (CNS) pinpointed LRP1 as the likely receptor facilitating the endocytosis of soluble tau forms, including monomers and oligomers [43]. Given these findings, it can be hypothesized that a similar mechanism may play a role in other prion-like proteins including α-syn. HSPGs and PFFs colocalize with each other, where seeding activity of α-syn was observed [25]. In another study using *C. elegans* models, α-syn PFFs were fed to worms resulting in the spread of pathology from the gut, while in HSPG gene knockdown worms, PFF aggregation was decreased, and dopamine neurons were protected from PFF toxicity suggesting that HSPGs play a role in α-syn seeding [44]. 

#### Cellular prion protein (PrP^c^)

PrP^c^ is a cell surface glycoprotein and a toxicity-transducing receptor for different misfolded protein isoforms. PrP^c^ is expressed in both neurons and glial cells and thus has the potential to facilitate glia-to-neuron transmission of α-syn. It uses the second charged cluster domain to actively bind to α-syn protofibrils [45]. Both in vivo and in vitro experiments have confirmed the behaviors of PrP^c^ interactions with α-syn. Increased binding of mouse α-syn protofibrils was observed in *Prnp*-transfected HEK293 cells. These results were corroborated by in vitro observations of the motor cortex of mice overexpressing PrP^c^. Increased transport of α-syn throughout different brain regions, including the striatum, substantia nigra, amygdala, and neocortex have also been observed with PrP^c^ overexpressing mice [46]. As a key mediator in α-syn-induced synaptic impairment, PrP^c^ forms a complex with extracellular α-syn oligomers and triggers the phosphorylation of Fyn kinase via metabotropic glutamate receptors. This engagement activates the NMDA receptor and alters calcium homeostasis, leading to synaptic and cognitive deficits. This mechanism supports the hypothesis that a receptor-mediated process, independent of pore formation and membrane leakage, triggers early synaptic damage induced by extracellular α-syn [47]. 

#### Transmembrane glycoprotein NMB (GPNMB)

GPNMB is highly expressed in various brain regions, including neurons, astrocytes, and mainly microglial cells, and its role in neuroinflammation and neurodegenerative diseases is complex, with evidence suggesting both anti-inflammatory and pro-inflammatory effects depending on the context and cell type [48, 49]. Recently, GPNMB has been identified as a risk locus for PD through several genome-wide association studies [50, 51]. Additionally, Diaz-Ortiz et al. showed that loss of GPNMB in iPSC-derived neurons led to effects on synaptic biology, prominently identifying α-syn as a central protein hub in network analyses of genes. Interactions between these proteins were further explored and α-syn internalization was observed in both iPSC neurons and HEK293 cell lines. Inhibiting GPNMB reduced α-syn uptake and internalization. In clinical analyses, GPNMB levels were elevated in the plasma of PD patients [52]. However, the results of GPNMB’s impact on α-syn pathology have been disputed. A study found that GPNMB levels were elevated in the substantia nigra of PD patients with decreased GCase activity and increased glycosphingolipids, whereas transgenic mice modeling synucleinopathy did not show these changes, suggesting that GPNMB elevation is related to lipid-induced degeneration rather than α-syn load [53]. Deleting the Gpnmb gene also had no significant impact on histological, cellular, behavioral, neurochemical, or gene expression outcomes in three different mouse models of neurological diseases, indicating that GPNMB may not play a major role in the development of pathology or functional defects in these models [54]. 

#### Summary of receptors

The examination of the cellular mechanisms involved in α-synucleinopathies reveals a complex network of proteins that facilitate the uptake and propagation of α-syn across cells, highlighting potential therapeutic targets. While receptors like GPNMB, LRP1, FcγRIIB, HSPGs, LAG3, and APLP1/LAG3 complex have been shown to contribute to α-syn internalization, other proteins [6, 9, 23, 25, 39, 52], including PrP^C^, integrin CD11b, α3-NKA, and TLR4, are known to propagate pathology by responding to α-syn signal and facilitating cellular damage [29, 34, 36, 47]. 

LRP1’s selective mediation of soluble α-syn uptake, alongside its role in brain microtubular function, establishes it as a potential factor in disease pathology [6]. Conversely, LAG3’s interaction with α-syn fibrils and its potential to exacerbate endothelial damage brings forward another avenue for therapeutic intervention [13]. The dual function of LAG3 and APLP1 in facilitating α-syn endocytosis, particularly when they form a complex, are crucial factors in the uptake of fibrillar α-syn [23]. The involvement of immune receptors like TLR4 underscores the inflammatory component of α-synucleinopathies, suggesting that modulation of neuroinflammation could be a strategic therapeutic focus [30, 31]. Meanwhile, integrin CD11b, which activates microglial responses, points to the multifaceted nature of α-syn pathology [36]. 

Future research should concentrate on disentangling the specific contributions and interactions of these molecules in the progression of α-synucleinopathies. Targeting LAG3 and the LAG3-APLP1 complex offers a promising therapeutic strategy for α-synucleinopathies due to several distinct advantages. These include the specificity of LAG3 for pathogenic α-syn fibrils [9]. Inhibiting the binding of α-syn to LAG3 could effectively impede the uptake and spread of these toxic aggregates in the brain. The LAG3-APLP1 complex further accentuates this effect by enhancing the endocytosis of α-syn, making the disruption of this complex a potentially powerful means to mitigate α-syn-associated toxicity [23]. Moreover, LAG3’s role as an immune receptor presents an opportunity to modulate inflammatory responses that contribute to disease progression. The flexibility of intervention offered by existing LAG3 antibodies, initially developed for cancer treatment [55], could expedite the development of neurodegenerative disease therapies. Importantly, the impact of LAG3 and APLP1 on cerebral vasculature suggests that targeting this complex could have benefits beyond neuron-focused approaches, addressing the broader neurovascular implications of α-synucleinopathies [13]. Studies indicating the neuroprotective potential following the inhibition or deletion of LAG3 provide additional support for this therapeutic target [9, 11]. Moving forward, it will be essential to unravel the molecular details of the LAG3-APLP1 and α-syn interactions and to understand potential compensatory pathways that might arise in response to such treatments. Ensuring that inhibiting the LAG3-APLP1 complex does not disrupt normal protein functions will be crucial for developing safe and effective therapies against α-synucleinopathies [23]. With these considerations in mind, LAG3 and the LAG3-APLP1 complex remain compelling targets for future research and therapeutic development.

### Membrane receptors and proteins as a therapeutic target

#### Expression patterns and therapeutic implications

To approach the therapeutic targeting of transmembrane receptors and proteins implicated in the propagation of α-synucleinopathies, understanding their expression patterns across various brain cell types is important. The expression levels of these proteins, as reported by the Human Protein Atlas [56], are summarized in Table 2. Neurons with high energy demands, such as dopaminergic neurons, are particularly susceptible to α-syn accumulation due to their extensive axonal networks and dependence on mitochondrial function [57]. The vulnerability of certain neurons, like those in the substantia nigra, may be due to a combination of structural features (i.e., large axonal networks) and functional stressors (i.e., mitochondrial dysfunction and redox imbalance). This susceptibility underscores the need to target both the cellular and molecular mechanisms that exacerbate α-syn pathology [57, 58]. Identifying which neuron subtypes are more prone to α-syn toxicity allows researchers to focus on specific membrane receptors and proteins that are either abundant or uniquely active in these neurons. These receptors might facilitate α-syn binding, uptake, and intracellular trafficking, thereby driving the spread of pathological α-syn across connected neural circuits.

While some of these receptors are predominantly neuronal, others are critical in glial cells, like astrocytes, microglia, and oligodendrocytes. Modulating microglial activity could influence disease progression in several ways. Although microglia are responsible for clearing misfolded α-syn through phagocytosis, their excessive or chronic activation can lead to an overproduction of pro-inflammatory cytokines, exacerbating neuroinflammation and neuronal damage [59]. Moreover, excess α-syn can promote increased microglial and neuronal uptake of α-syn, creating a cycle of microglial activation, oxidative stress, conversion of astrocytes to neurotoxic reactive astrocytes, and neuron death [58, 59]. Therapies targeting TLR4 might strike a balance between promoting α-syn clearance and reducing harmful inflammation [30, 31]. Glial cells can also uptake α-syn from neurons, process it, or release it, contributing to further spread [59]. Interventions targeting glial cell receptors and preventing cross-talk between glial cells and neurons could be therapeutic, reducing neuronal damage and α-syn propagation mediated by inflammatory responses [32]. 

It is also important to recognize that some receptors, like LAG3 and FcγRIIB, are expressed at low levels in the human brain according to the Human Protein Atlas [56], yet research has found functional evidence for their roles in neurodegeneration and their presence in brain samples [16, 60]. This discrepancy highlights the necessity for additional validation techniques, particularly for proteins with low expression levels or those transiently upregulated under pathological conditions. Further investigation into the functional consequences of these proteins is crucial, as their biological significance might not always correlate with their baseline expression levels. For instance, transient upregulation during neuroinflammatory responses might significantly impact disease progression, emphasizing the need for targeted investigations into these proteins’ roles in neurodegenerative processes [61, 62]. Comprehensive profiling and evaluation of the pathological impact of these proteins are essential for guiding the design and application of targeted interventions effectively.

#### Current targeted therapies

**Fig. 1:**
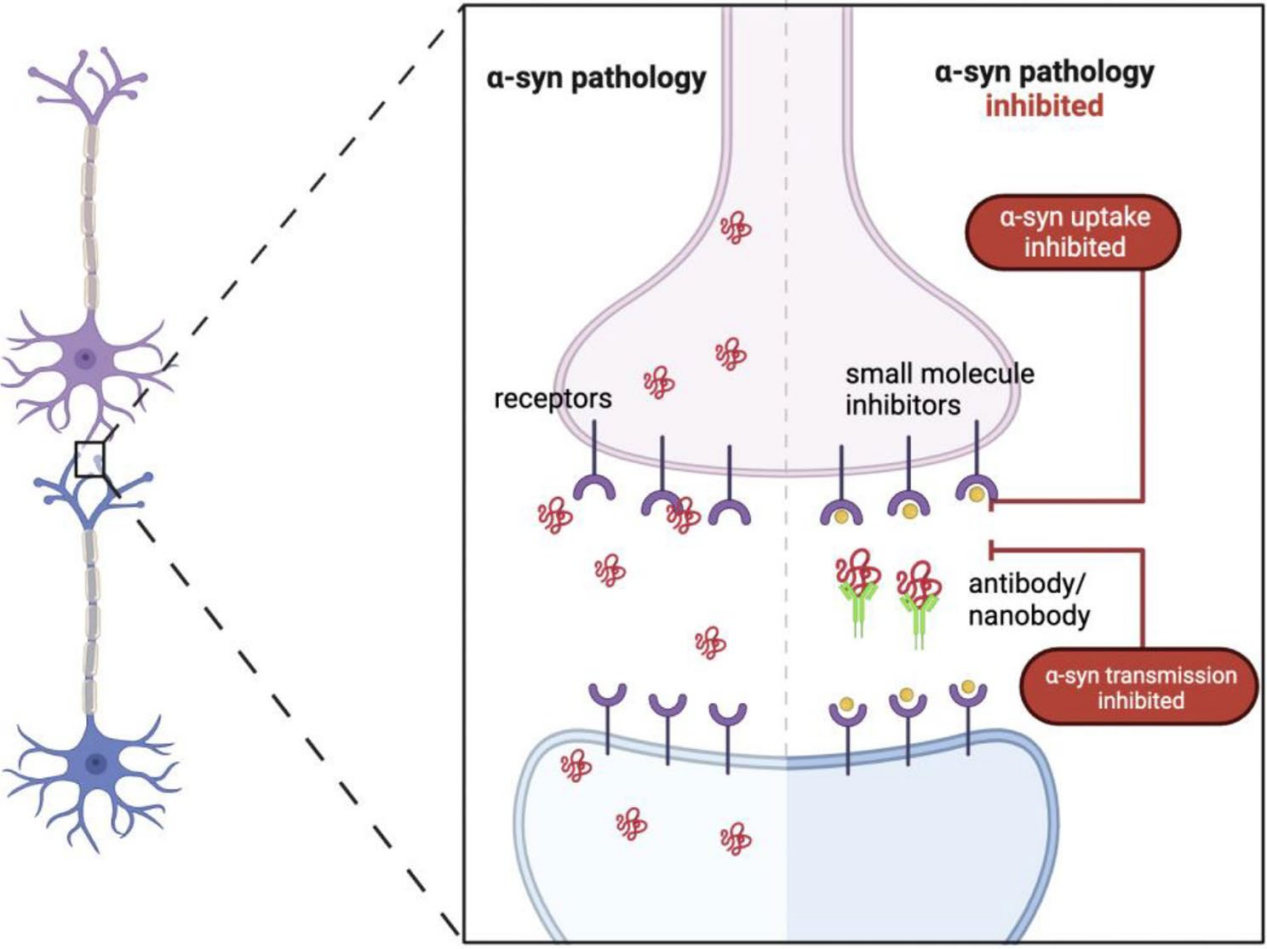
Pathological transmission of α-syn blocked by small molecule inhibitors, antibodies, and nanobodies. The right illustrates bindings of small molecules to specific cell-surface receptors of α-syn, inhibiting α-syn binding and internalization. Extracellular α-syn aggregates can be limited and reduced by exogenously introduced antibodies and nanobodies, providing viable therapeutics to halting neurodegeneration. The left demonstrates unregulated α-syn transmission from cell to cell [9, 64–66].

Therapeutics targeting α-syn binding proteins and cellular receptors currently are limited to antibodies and are illustrated in Fig. 1 [63–65]. Antibodies play important roles in research, diagnosis, and treatment. As part of the immune system, they can intervene with targets in the extracellular space and at the cell membrane [66]. For many of the targets’ above, limited research has gone into assessing the efficacy and feasibility of using antibodies as therapeutics. Anti-NKA antibodies showed efficacy where an NKA-stabilizing monoclonal antibody targeting the extracellular region ^897^DVEDSYGQQWTYEQR^911^ (DR region) of NKAα subunits demonstrated reduced pathologic α-syn, improvements in learning and memory, as well as neuromotor performances in in vivo PFFs models of mice [64]. LAG3-targeted therapies have become increasingly popular; many are in clinical trials for cancer. These therapies include monoclonal antibodies, soluble LAG3-immunoglobulin fusion proteins, and anti-LAG3 bispecific drugs [65]. Mao et al. showed that two different anti-LAG3 antibodies, C9B7W and 410C9, reduced α-syn PFFs pathology [9]. 

Screening campaigns for small molecule inhibitors of pathologic α-syn uptake to prevent α-syn accumulation and pathology hold promise [63]. Small molecules agonists and antagonists are yet to be fully exploited and integrated into targeting membrane receptors and proteins in α-syn pathology research. Together, both antibodies and small molecule approaches to blocking pathologic α-syn uptake should be favorably looked upon as potential therapeutics [63–65].

## Targeting cell to cell transmission

### Cell to cell transmission of α-synuclein in parkinson’s disease

The concept of cell-to-cell transmission of α-syn pathology in PD has been supported by a growing body of evidence from human postmortem and grafted neurons, epidemiological, and rodent studies. The Braak hypothesis, first proposed nearly two decades ago, posits that α-syn aggregation initiates in specific brain regions and progressively spreads throughout the CNS, impacting particular neuron populations. This model relied on the idea that pathologic α-syn can move between cells, inducing pathological changes in the recipient cells. According to this hypothesis, sporadic PD might originate in neurons of the nasal cavity and gut before spreading to the CNS, with the spinal cord involved at a later stage [67, 68]. This hypothesis is supported by postmortem studies in PD patients with healthy neurons grafted into their brains. These grafted neurons develop α-syn and ubiquitin-positive Lewy bodies over time, suggesting that misfolded α-syn can propagate between cells [69, 70]. For instance, a patient who received unilateral transplantation of embryonic dopaminergic neurons in the putamen showed ubiquitin- and α-syn-positive inclusions in 11–12% of the grafted neurons, indicative of pathologic transmission from the host brain to the transplants [71]. Epidemiological evidence provides further support as nonmotor symptoms like hyposmia and constipation often precede motor symptoms by years, linked to the early accumulation of α-syn in the enteric nervous system and olfactory bulb consistent with the notion that pathology spreads from the gut to the brain [72–76]. In mouse models injecting α-syn PFFs into the gut and brain initiates the spread of α-syn pathology, first affecting the enteric nervous system and directly through the CNS, then progressing to connected brain regions [77–79]. Enteric expression of asparaginyl endopeptidase c-terminally truncated α-syn leads to the spread of pathologic α-syn into the CNS providing support for the idea that the spread of pathologic α-syn is an intrinsic pathogenic mechanism occurring in PD and related α-synucleinopathies [80, 81]. Additionally, a single injection of synthetic α-syn fibrils into the striatum of WT, non-transgenic mice triggered the spread of pathogenic α-syn through cell-to-cell transmission, resulting in Parkinson’s-like Lewy pathology in interconnected brain regions [82]. Even without endogenous mouse α-syn, injecting human WT α-syn enables prion-like propagation of α-syn pathology in mice, with intraneuronal inclusion bodies spreading to the contralateral hemisphere and rostral and caudal areas [83]. 

Despite the evidence supporting prion-like spreading and cell-to-cell transmission of pathologic α-syn, this idea has faced criticism. For instance, not all PD cases follow Braak’s staging system, with only about half of patients showing the proposed spread pattern from the gut or olfactory bulb to the brain [84]. Some patients exhibit Lewy pathology in higher brain regions without corresponding pathology in the dorsal motor nucleus of the vagus, challenging the uniformity of this spread [85]. However, recent studies suggest that there is Body First and Brain First PD, which likely accounts for these discrepancies [58, 86]. Evidence suggests that cell-autonomous and region-autonomous mechanisms contribute to PD pathogenesis, with certain neurons inherently more susceptible to degeneration due to their unique cellular traits [57]. For instance, the substantia nigra’s dopaminergic neurons are particularly vulnerable due to high mitochondrial stress, extensive axonal networks, and calcium-dependent energy demands, which might not be solely explained by α-syn propagation [57]. Consistent with this notion is the observation that adeno-associated virus (AAV) overexpression of A53T α-syn leads to severe degeneration in substantia nigra dopaminergic neurons, while sparing ventral tegmental area neurons, underscoring inherent differences in vulnerability and potentially undermining the cell-to-cell transmission hypothesis of pathologic α-syn [87]. Vulnerable neurons, such as those in the substantia nigra pars compacta, often exhibit high basal mitochondrial oxidant stress [88], extensive axonal arbors [89], and calcium-dependent bioenergetic demands [90], which predispose them to degeneration. It is now becoming clear that some neurons are relatively resistant to the toxic effects of pathologic α-syn and that they serve as conduits for the spread of pathologic α-syn along neuronal circuits. These intrinsic traits in different neurons likely interact with α-syn pathology, creating ‘tipping points’ that lead to vulnerability in some neurons, while enabling survival in others that act as conduits for its spread [58, 91]. Thus, elucidating the mechanisms by which pathologic α-syn causes differential vulnerability to spread is crucial for future treatments focusing on preserving healthy neurons and delaying the onset of motor and nonmotor symptoms.

### Extracellular release of α-syn

Understanding the mechanisms behind the release of α-syn to the extracellular space is critical in further delineating the intercellular propagation of α-syn. Both passive and active transport are possible in the secretory pathways involved in cell-to-cell spreading.

Despite a signal sequence for secretion, α-syn can permeate the cell membrane and is detectable in cerebrospinal fluid, plasma, and cell culture supernatants [92]. This permeation is primarily facilitated through passive diffusion, a process distinctive to its monomeric and small oligomeric forms, rather than its aggregated states [93]. Research highlights a nuanced mechanism where α-syn oligomers, upon adopting ring-like coiled structures from α-helical conformations, penetrate cell membranes swiftly [94]. These oligomers initially attach to and fold on the membrane surface, transitioning into coiled and β-strands formations, and with additional monomers, can form large pore-like oligomers that lead to cell membrane perforation [95, 96]. Furthermore, while externally added α-syn monomers can freely enter and exit cells through diffusion, α-syn that is internally synthesized is likely to be secreted, a possible consequence of post-translational modifications [97]. 

Active transport of α-syn across the cell membrane into the extracellular matrix can happen through endoplasmic reticulum (ER)-Golgi-dependent exocytosis, non-classical exocytosis, and association with vesicles [98–101]. In ER-Golgi-dependent exocytosis, USP19 aids in the secretion of misfolded α-syn species by removing ubiquitin and facilitating their export outside the cell through late endosomes. This process, crucial for cellular quality control, involves the collaboration of USP19 with Heat shock protein 90, DNAJC5, and Hsc70, forming a complex that enables the expulsion of α-syn aggregates into the extracellular space [102, 103]. Interestingly, for non-classical endocytosis, α-syn translocation and secretion in vesicles is increased for abnormal α-syn species [100]. Furthermore, α-syn aggregates within multivesicular bodies-derived exosomes are expelled through a calcium-dependent mechanism and have demonstrated an increased seeding activity in the cerebrospinal fluid of patients with synucleinopathies compared to controls [101]. Cell culture experiments indicate that α-syn aggregates can be efficiently transferred from SH-SY5Y cells to healthy cells via exosomes, a process exacerbated by lysosomal dysfunction, such as that induced by bafilomycin A1 treatment [99]. 

Tunneling nanotubes (TNTs) are thin membranous channels lined with F-actin that connect cells. Many proteins and nucleotides, including endosomes, lysosomes, and mitochondria species are known to travel between cells using TNTs [104]. Concerning α-synucleinopathies, TNTs bridge the cytoplasm of neighboring cells, allowing transfer of α-syn species between neurons and non-neuronal cells [105]. Research suggests that there is increased formation of TNTs due to α-syn fibrils, which is speculated to be one of the cell’s ways of dealing with stress by connecting to unstressed cells [105]. Intercellular transfer of α-syn through TNTs is highly efficient in both neuronal-like cells and in primary neurons, with 100% of acceptor cells containing α-syn and 38% of α-syn puncta transferred between donor and acceptor cells in one study [105]. This formation of TNTs is observed in both SH-SY5Y cells and primary brain pericytes for α-syn transfer and long-distance electrical coupling [106]. Further studies demonstrated α-syn transfer between human neuronal precursor cells in a cell-to-cell contact-dependent manner through TNT-like structures [107]. The exact mechanism by which TNTs are able to propagate cell-to-cell transmission of α-syn species remains elusive. α-Syn fibrils are found in vesicles after transfer from donor cells, suggesting that α-syn was carried by lysosomal vesicles through the lumen of the TNTs [105]. Further research discovered that the transfer of vesicles is regulated by Wnt/Ca^2+^ pathway activation and specifically modulated by the β isoform Ca^2+^/calmodulin-dependent protein kinase II in both CAD cells and primary neurons [108]. Regulating TNT activity thus provides a potential opportunity for inhibiting the cell-to-cell propagation of α-syn transfer.

### Uptake of α-syn

The uptake of α-syn in neurons and glial cells happens through both passive and active mechanisms. For α-syn monomers, diffusion into cells happens readily without the need for endocytosis or regulated temperatures. However, bigger molecules, particularly oligomeric and fibrillar α-syn utilize intracellular components to facilitate dynamin-dependent endocytosis [109]. 

The N-terminal domain and the hydrophobic NAC domain are responsible for the translocation of α-syn across the plasma membrane, as demonstrated in experiments using HeLa cells, neuronal cells, hematopoietic cells, and Chinese hamster ovary cells. As the diffusion of α-syn is modulated by sequence motifs on the protein itself, α-syn may bind to non-specific cell types. The N-terminal domain of α-syn has high affinity for lipid layers as well [110]. The translocation process happens when toxic α-syn oligomers bind and perturb the cellular membrane by inserting into and disrupting the lipid bilayer [111]. Perturbation of cellular membrane by α-syn can happen in many ways, including amyloid pore-like channel formation, membrane thinning, stripping, or tubule formation [112, 113]. The uptake process not only introduces α-syn seeds into recipient cells, but α-syn can also aggregate and form larger fibrillar structures [114]. In both neuroblastoma cells and primary cortical neurons, researchers have successfully reduced toxicity of α-syn oligomers by mutating regions that strongly interact with membranes [111]. 

Most α-syn aggregates are internalized into recipient cells by classical endocytosis and are able to interact with intracellular α-syn monomers, driving aggregation and promoting pathology [115]. Early studies have proposed clathrin-mediated endocytosis as the mechanism for α-syn uptake in cells. Protein accumulation on the plasma membrane and subsequent internalization into specific Rab-positive vesicles for sorting and degradation have been identified. Rab proteins, which are small GTPases, play a crucial role in the regulation of protein transport and compartmentalization along endocytic and exocytic pathways, facilitating α-syn’s trafficking within the cell. Extracellular α-syn follows a pathway similar to transferrin during internalization, ultimately leading to its degradation via Rab7-positive vesicles in lysosomes to prevent intracellular accumulation [116]. Cell surface receptors play a crucial role in the uptake of extracellular α-syn as well by not only binding but also internalizing α-syn. In a recent study, Mao et al. revealed that LAG3 and APLP1 collaborate in facilitating the uptake of α-syn PFFs via endocytosis, thereby promoting the propagation and spread of pathology. Genetic and antibody-based inhibition of this protein interaction significantly reduces endocytosis, underscoring the critical role of targeting cell surface receptors as a strategy to reduce pathological load. Research has also shown that reducing temperatures and applying endocytosis inhibitors can diminish the uptake of α-syn in neuronal cells, potentially mitigating its toxicity [117]. 

Uptake of α-syn through exosomes is possible through direct fusion of the exosome containing α-syn with the plasma membrane. This entry pathway has been demonstrated in human H4 cells, primary cortical neurons, and human neuroblastoma cells with α-syn oligomers [115]. 

### Targeting cell-to-cell transmission as a therapeutic target

Targeting cell-to-cell transmission mechanisms for therapeutic intervention in the context of α -synucleinopathies, while promising in theory, faces significant challenges that may limit its viability as a therapeutic approach. This is reflected through the lack of research using these intercellular transmission pathways as intervention opportunities. Many of the intervention strategies to target different methods of intercellular spread are illustrated in Table 3.

The research by Dilsizoglu Senol et al. investigated the viability of pharmacologically targeting TNTs to slow the seeding and propagation of α-syn. The study evaluated the effect of tolytoxin, a cyanobacterial macrolide known to disrupt actin dynamics, on the formation of TNTs and the intercellular transfer of α-syn fibrils and mitochondria. The findings revealed that tolytoxin, at specific nanomolar concentrations, was able to reduce both the number of TNTs and the transfer of α-syn fibrils between SW13 and SH-SY5Y cells, as well as the number of α-syn puncta within the cells [118]. However, the research also highlighted a significant therapeutic challenge: while tolytoxin reduced the pathological transfer of α-syn fibrils, it also impaired the transfer of healthy mitochondria between cells. This is a critical issue because the transfer of mitochondria is an important aspect of cellular homeostasis and defense mechanisms, particularly in the brain where neurons depend on healthy mitochondrial function for survival and maintenance. Therapeutics that interfere with this process could, therefore, have detrimental effects on neuronal health and overall brain function.

Additionally, the multiplicity of pathways involved in α-syn transfer using both passive and active transport might allow for various opportunities for interventions, but also inhibiting one specific pathway might not be sufficiently effective. In a study using the dominant negative mutant of dynamin-1 K44A, which is GTPase inactive, researchers confirmed that the expression of this mutant resulted in an endocytosis blockade. The expression of dynamin-1 K44A led to α-syn accumulation on the cell surface, indicating that the fibrils were unable to be internalized via the blocked endocytic pathway. While this mutation can reduce the uptake of aggregated α-syn in cultured cells, the internalization of monomeric α-syn was not affected [93]. This result suggests that α-syn may use alternative pathways, such as direct membrane translocation, for cell entry. Hence, therapies that aim to target the endocytosis and exocytosis of α-syn by inhibiting one specific pathway might not be sufficiently effective, since the protein could utilize other mechanisms to enter cells. Many cellular functions rely on intercellular pathways to communicate between cells. Vital cellular processes are at risk of adverse effects if therapeutics are not specific enough to target just the α-syn transmission pathways.

## Direct targeting of pathological α-synuclein

### α-Synuclein pathology

α-Syn aggregates are in of themselves a therapeutic target. Various stages, ranging from the encoding of the α-syn gene in DNA to the aggregation of α-syn into fibrils, can be strategically addressed and interventions designed. The imbalance of α-syn synthesis, aggregation, and clearance informs the nature of disease progression in the CNS. Cell-autonomous effects of α-syn on neuronal dysfunction and vulnerability are central to its pathology. α-Syn aggregates within neurons, primarily in dopaminergic cells of the substantia nigra for PD, leading to cellular stress, mitochondrial dysfunction, impaired protein degradation, and synaptic dysfunction. These aggregates interfere with vesicle trafficking, affect synaptic function, and promote oxidative stress, making neurons more vulnerable to degeneration [119]. Affected pathways, such as those involving mitochondrial dysfunction, impaired autophagy-lysosomal pathways, and proteasomal degradation, contribute to cellular stress and eventually trigger mechanisms of neuronal death. These pathways may also influence the cellular release of α-syn, especially as the normal cellular machinery becomes overwhelmed. Stress responses, such as exocytosis of misfolded proteins or vesicle-mediated secretion, can lead to the release of α-syn into the extracellular space [120–123]. Additionally, research suggests that α-syn pathology spreads through anatomically connected neuronal circuits, with the pattern of spreading modulated by factors like neuronal connectivity and endogenous α-syn expression levels. Early synaptic dysfunction and network disruption, coupled with neuronal hyperactivity, significantly promote the aggregation and propagation of α-syn pathology, suggesting that both cell-autonomous factors and trans-synaptic spread can influence disease progression [124, 125]. 

The levels of α-syn can be inherently increased due to gene variants such as the polymorphism known as Rep1 [126]. Correspondingly, the human system has innate clearance systems that can offset this imbalance using direct proteolysis, the proteasome, autophagy, and binding to molecular chaperones [127, 128]. The native state of α-syn exists as unfolded monomers while some alternative hypotheses believe its form in the cytosol is of a tetrameric α-helical oligomer resistant to aggregation [129]. Researchers have yet to uncover the exact mechanistic pathway in which oligomers assemble into aggregated fibrils [130]. Studies indicate α-syn aggregates intracellularly, but it can also exist in the extracellular matrix. In fact, its toxicity in cell-to-cell transmission is seen in its role outside the cell, where misfolded seeds of α-syn are taken up by adjacent cells leading to misfolding of native α-syn in the recipient cell [131]. To further characterize spreading, a study revealed that in the pedunculopontine nucleus, only cholinergic neurons developed significant α-syn pathology after being inoculated with α-syn PFFs, indicating that the type of neuron influences the uptake and retention of α-syn. Additionally, while synaptic connections facilitated the spread of α-syn pathology, the extent of spread was not directly correlated with the strength of these connections, and many brain regions were capable of clearing the induced pathology over time, suggesting a complex interplay of factors in the spread of pathological α-syn [91]. Table 4 summarizes key steps along the way targeting the formation of α-syn fibrils to further prevent cell-to-cell spreading.

### Pathological α-synuclein as a therapeutic target

#### Gene silencing via RNAi and antisense oligonucleotides

**Fig. 2:**
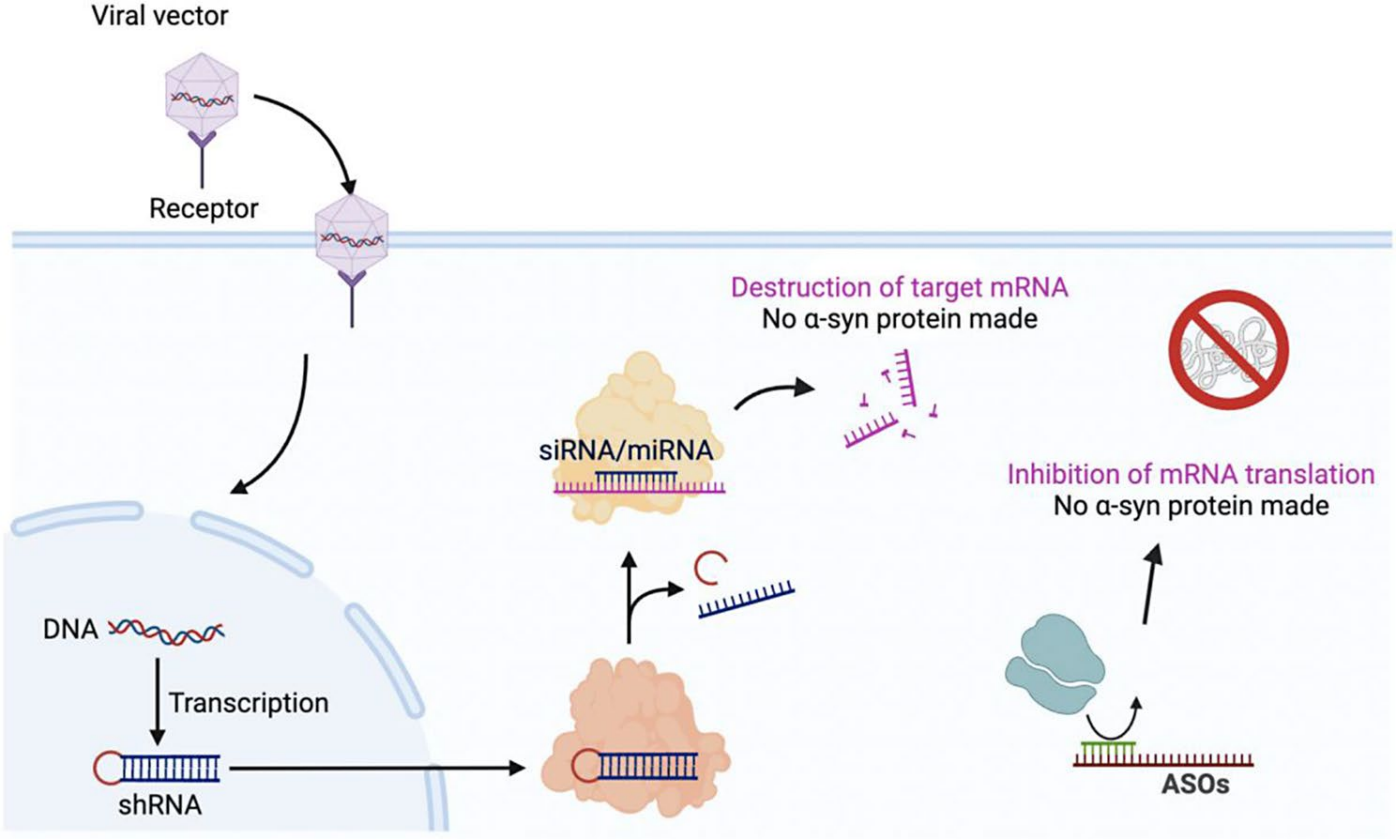
Gene silencing approaches to inhibit α-syn protein translation. Viral vectors can introduce shRNAs into the cell where they are being processed into miRNAs or siRNAs. These non-coding RNAs are guided to the target mRNA, which encodes α-syn, thereby degrading the mRNA before any α-syn can be synthesized. Further, ASOs can also be introduced into the cell, where they bind with target α-syn mRNA and prevent the mRNA from being further translated into protein. These therapeutics have the potential to reverse disease progression by targeting α-synucleinopathies at its genetic level [132, 133, 136, 138–141].

Understanding the pathway from native α-syn monomers to fibrilizations that lead to α-syn neurotoxicity can allow for therapeutic interventions that target specific steps within this process. Targeting the gene encoding α-syn introduces permanent change at the genetic level of DNA and is at the forefront in strategies to halt α-synucleinopathies and is illustrated in Fig. 2 [132]. In the past few years, there have been numerous approaches employed to silence the SNCA gene. One approach involves using a ribozyme to down-regulate SNCA expression, while another approach utilizes RNA interference (RNAi) to interfere with the expression of WT or mutant SNCA [132]. When using strategies such as RNAi for therapeutic intentions, it is crucial to ensure that the silencing vectors used are not harmful to dopaminergic neurons [133]. Multiple reports of various RNAi delivery methods have resulted in decreased cell viability as well as dopaminergic neuron death [1]. One study compared the efficacy of using short hairpin RNA (shRNA) embedded in a microRNA (miRNA) backbone and driven by a pol II promoter with shRNA that was not embedded in a miRNA backbone but driven by a pol III promoter. Results displayed significantly lower toxicity for the miRNA-embedded silencing vector [132]. A prior study developing a dual cassette lentiviral vector for delivering shRNA targeting α-syn showed effective gene silencing in vitro and in vivo. The lentiviral-mediated RNAi successfully silenced human α-syn in rat brains, leading to reductions in α-syn levels and amelioration of motor deficits without causing neurodegeneration [133]. Similarly, Kim et al. identified the RNAi sequence miSyn4, which demonstrated the highest efficiency in knocking down α-syn. Injecting AAV vectors expressing miSyn4 into Thy1-hSNCA mice significantly reduced α-syn levels in both the midbrain and cortex and ameliorated behavioral deficits without causing adverse effects on neurons or glial cells [134]. A more recent study demonstrated that long-term RNAi knockdown of α-syn in the adult rat substantia nigra reduced neurodegenerative changes without significant side effects [135]. However, the low penetrance of the blood-brain barrier makes the delivery of viral vectors a significant challenge [136]. In another effort to improve delivery efficiency, polyethyleneimine F25-LMW was complexed with siRNA. Not only was SNCA mRNA and protein reduced by more than half, the mice showed no adverse effects [137]. There has been recent progress in the noninvasive delivery methods of α-syn gene silencing vectors using magnetic resonance-guided focused ultrasound. Combined with microbubbles, this method can increase the permeability of the blood-brain barrier both locally and transiently for several hours [138]. In another recent approach, rather than binding to the α-syn protein itself, the study capitalized on the ordered structures in the 5′ untranslated region of the SNCA mRNA. They used Synucleozid-2.0, a small molecule that inhibits α-syn translation by binding to the structured 5′ UTR, and further developed it into a RiboTAC (Syn-RiboTAC), demonstrating selective degradation of SNCA mRNA as well as offering enhanced cytoprotective effects [139]. Indeed, gene silencing may hold promising features to stop α-syn propagation, but several limitations still apply and are yet to be solved.

Antisense oligonucleotides (ASOs) are short, synthetic pieces of DNA or RNA that can selectively bind to specific mRNA molecules. By binding to the mRNA, ASOs can modulate gene expression and protein production [140]. In the context of α-synucleinopathies, ASOs can target intracellular SNCA RNA which reduces all forms of α-syn [141]. This disease-modifying therapeutic has been explored in human cell lines, human α-syn transgenic mouse models, and non-human primates. ASOs were administered and observed to lower SNCA production and decrease α-syn levels even with pathology already present. This key finding offers a potential for reversing neurodegeneration [141, 142]. Additional ASO approaches include exosome-mediated delivery; exo-ASO4 delivered via exosomes significantly attenuated α-syn aggregation, reduced neurodegeneration, and improved locomotor functions in transgenic PD mice [143]. An amido-bridged nucleic acids modified ASO has also shown efficacy in downregulating SNCA mRNA and protein while ameliorating neurological defects in PD mice model [144]. In another mouse model of PD, local injection of ASOs against SNCA mRNA into the striatum significantly reduced endogenous α-syn levels, thereby preventing and inhibiting the spread of phosphorylated α-syn pathologies throughout the brain, including the contralateral hemisphere. This demonstrates the potential of ASO treatment to mitigate neurodegenerative progression even when administered after the initial pathology onset [145]. Table 5 summarizes studies related to gene silencing targeting α-syn in animal models [141]. 

#### Small molecules inhibitors

Small molecules have been discovered to inhibit or revert the aggregation and propagation of α-syn [146–149]. One research study using a *C. elegans* model exhibiting early motor dysfunction caused by α-syn has revealed the potential of small molecules in preventing α-syn oligomerization. By combining the model with a drug repurposing screen, several promising candidates were identified. Notably, rifabutin, known for its ability to penetrate the blood-brain barrier, demonstrated effectiveness in reducing extracellular α-syn. However, further research needs to be conducted on the efficacy and mechanism of its therapeutic effects [146]. Another study identified SynuClean-D (SC-D), a low-toxicity and cell-permeable small molecule, to be able to disrupt the assembly of α-syn fibrils. SC-D also shows potential in reducing α-syn seeding which can limit soluble α-syn from turning into its insoluble form [147]. The recent discovery of the iridoid genipin showed that it was able to decrease fibrillar formation of α-syn. By impacting the structure of α-syn, genipin also affects cellular processes including energy metabolism, lipid storage, or vesicular trafficking of α-syn. In a *Drosophila* PD model, genipin increased lifespan and improved motor capabilities of the flies expressing α-syn [150]. Most recently, the inhibitor 03A10 was discovered to bind to α-syn aggregates and exemplified therapeutic effects in the olfactory bulb and substantia nigra by decreasing α-syn accumulation and inflammation in an MPTP mouse model [149]. Some small molecules have entered clinical trials. The small molecule Anle138b is able to reverse motor function to healthy controls in mice overexpressing human α-syn. Anle138b binds to oligomers which alters its structure and neurotoxic properties, depleting pre-amyloid oligomers and impairing fibril growth [148]. It has successfully finished Phase 1a trial where it was found to be safe and and tolerable for healthy participants [151]. In its Phase 1/1b clinical study, its safety and tolerability were deemed acceptable with a Phase 2 study underway [152]. 

#### Antibodies and Nanobodies

Recent discoveries of antibodies and nanobodies that can bind exclusively to α-syn fibrils have presented yet another therapeutic strategy and is illustrated in Fig. 1 [147, 153]. Both passive and active immunizations have entered clinical trials as shown in Table 6. Passive immunization offers several advantages as a treatment option due to its ability to control different factors, such as binding affinities and antibody concentration. It also allows for quick intervention in case of side effects and can bypass T-cell stimulations. Despite the need for frequent administration, these benefits make passive immunization a favorable therapy [154]. Passive immunization techniques involve antibodies directly binding to the different domains of α-syn [155]. 

In recent developments, several monoclonal antibodies have been explored for their potential to target α-syn aggregates. ABBV-0805, a monoclonal antibody with selective affinity for oligomeric α-syn, showed promise in mouse models by reducing α-syn aggregates and prolonging survival, leading to a Phase 1 clinical trial [156]. Although intravenous infusions in healthy subjects demonstrated tolerability and safety with a favorable immunogenicity profile of the drug and support for Phase 2 development, the sponsor, AbbVie withdrew ABBV-0805 for unknown reasons [157, 158]. Lu AF82422, a monoclonal antibody targeting all major forms of extracellular α-syn, is being investigated for its potential to slow disease progression in neurodegenerative disorders such as PD and MSA. In Phase 1 clinical trial, Lu AF82422 demonstrated safety, tolerability, and dose-proportional pharmacokinetics in both healthy participants and PD patients. The antibody showed target engagement by lowering free α-syn levels in the plasma and cerebrospinal fluid [159]. In the AMULET Phase 2 trial for MSA, Lu AF82422 exhibited a trend towards slowing clinical progression and reducing MRI volumetric loss, though the results were not statistically significant. Overall, Lu AF82422 was well tolerated, supporting further clinical development [160]. Another α-syn antibody, UCB7853, targeting α-syn aggregates, finished its Phase 1 clinical trial. The results of the UCB7853 trial are pending [161]. 

Additional antibodies include Biogen’s Cinpanemab (BIIB054) targeting the N-terminal of α-syn and was shown to inhibit α-syn spread and aggregation in preclinical studies; however, despite an acceptable safety profile in its Phase 1 trial [162], its development was discontinued after a Phase 2 study demonstrated no differences between treatment and placebo groups in the progression of motor function, nonmotor function, activities of daily living, quality of life, and imaging biomarkers at 52 weeks [163]. Prasinezumab (PRX002/RG7935), targeting the C-terminus of α-syn, showed safety and tolerability in a Phase 1 trial as well as binding of peripheral α-syn and success in a dose-dependent increase in the levels of prasinezumab in cerebrospinal fluid. Decreased α-syn in free serum after single infusion at the highest dose was also observed [164]. However, in its Phase 2 trial, there were no differences from baseline for the primary endpoint of the study after 52 weeks, demonstrating no effect on PD progression compared with placebo. Infusion reactions were also identified to be a main adverse event [165]. A recent post hoc analysis of subgroups who had faster progression of PD motor degeneration demonstrated that prasinezumab may be effective in slowing the progression of motor signs [166]. Therefore, prasinezumab is currently undergoing Phase 2b clinical trial to further assess the drug efficacy in PD patients with more advanced symptoms. With the exception of the prasinezumab post hoc analysis, the negative outcomes of these antibody clinical trials might suggest that targeting α-syn aggregates may not be an effective therapeutic route. Future studies are required to answer the effectiveness of passive immunization.

Active immunization has shown progress through the development of vaccines targeting α-syn pathology. AFFITOPE^®^ PD01A and PD03A are peptide-based vaccines designed to mimic an epitope in the C-terminal region of α-syn, aiming to elicit specific anti-α-syn antibodies without inducing a T cell response. PD01A was deemed safe after Phase 1 clinical trial, with antibody titers increasing significantly after booster immunization [167]. Shortly after, Phase 1 trial of PD03A was conducted and its safety and tolerability were found to be acceptable, the effect of a booster dose was not observed [168]. Both of these vaccines were assessed in another Phase 1 trial with MSA patients. Results indicated that the PD01A vaccine generated a stronger immune response, with 89% of treated patients showing α-syn-reactive IgG, compared to 58% for PD03A, while safety outcomes were consistent with previous trials [169]. There are plans for both vaccines in additional clinical trials. Similarly, UB-312, a vaccine developed by Vaxxinity, Inc, which links the C-terminal epitope of α-syn to a T helper peptide, has entered clinical trials. Phase 1 trial observed induced α-syn-specific antibodies in serum and cerespinal fluid of healthy participants while maintaining a favorable safety profile. A subsequent Phase 1b study for PD participants is currently ongoing with completion expected in 2025 [170]. 

While antibodies have a very limited ability to cross the blood-brain barrier, nanobodies present a promising alternative. Derived from camelid heavy-chain antibodies or engineered single-domain antibodies, nanobodies are small, soluble, and stable and preferred for intracellular targets as compared to antibodies that can only target extracellular α-syn [171]. VH14PEST is a human single-domain intrabody designed to target the NAC hydrophobic region of α-syn, which is essential for its initial aggregation. By binding to this region, VH14PEST disrupts aggregation and promotes the proteasomal degradation of monomeric α-syn. This mechanism not only reduces toxic aggregates but also helps preserve dopaminergic function in the striatum [172]. Additionally, previous nanobodies, most notably NbSyn2 and NbSyn87 [173], were shown to bind to both monomeric and fibrillar forms of α-syn. However, their efficacies are not well established; targeting total α-syn may alter the function of non-toxic α-syn monomers and prove less effective against toxic α-syn fibrils due to competitive binding [174]. As a result, recent fibril-specific nanobodies have been developed by researchers. Nbα-syn01 and its bivalent form were discovered to target the N-terminal of α-syn and show higher affinity for α-syn fibrils. Another recent study identified PFFNB2 to target intracellular aggregates. PFFNB2 effectively disintegrated α-syn fibrils and inhibited α-syn pathology from spreading to the cortex of transgenic mice expressing α-syn through intrastriatal-PFFs injection [174]. The development and application of antibodies and nanobodies for α-syn therapeutics are still in the early stages, requiring significant further research and clinical trials before they can be effectively administered as treatments.

### Challeneges and future prospectives

The complex pathology of α-syn engages multiple pathways and cellular constituents, which either potentiate or mediate the aggregation and propagation of α-syn fibrils. This review offers an examination of some of the targeted sites for α-syn spread, including cellular membrane receptors and proteins, intercellular transfer of α-syn, and pathologic α-syn. The review emphasizes advancements made over the previous five years, thereby encapsulating the current research landscape.

While much progress has been made in understanding α-syn aggregation and propagation, significant gaps in knowledge still limit the development of effective therapies. One of the greatest challenges in treating α-synucleinopathies is the overlap in molecular and pathological features among different neurodegenerative diseases [175]. For instance, α-syn aggregates are present in PD, DLB, and MSA, yet these diseases manifest with distinct clinical symptoms [176]. The lack of disease-specific biomarkers for differentiating between these conditions complicates diagnosis and treatment development [177]. Biomarker research, particularly in identifying disease-specific α-syn conformations or post-translational modifications, is urgently needed [178]. The influence of environmental factors on α-syn aggregation and pathology is poorly understood as well [179]. The variability in disease onset and progression may also be influenced by environmental factors, such as exposure to pesticides, heavy metals, or viral infections, but the mechanisms by which they modulate α-syn pathology remain unclear [180]. Another major unresolved question in neurodegeneration research is why certain diseases, like PD and LBD, primarily affect neurons, while others, such as MSA, primarily impact oligodendrocytes. The pathological hallmark of PD and LBD is the presence of Lewy bodies—aggregates of α-syn—within neurons, particularly dopaminergic neurons in the substantia nigra [181]. In contrast, in MSA, α-syn primarily accumulates in oligodendrocytes, the glial cells responsible for myelinating axons in the CNS [182]. This cell-type-specific vulnerability to α-syn aggregation is poorly understood, and understanding this distinction is critical for developing more targeted therapies for neurodegenerative diseases. Finally, the precise mechanisms through which α-syn is released from cells remain elusive. Although exocytosis and vesicle-mediated secretion are likely contributors, understanding the triggers for these processes is crucial [183]. One hypothesis suggests that cellular stress or autophagic-lysosomal dysfunction may drive the release of α-syn aggregates into the extracellular space, but this requires further validation [99, 184]. Without clarity on this release mechanism, therapeutic strategies that aim to mitigate α-syn transmission might not target the most effective points of intervention.

In this review, we have explored the pathology of α-syn both intracellularly and the mechanisms underlying its release and spread. An effective therapeutic strategy will likely require targeting both intracellular and extracellular α-syn. Given α-syn’s complex role in cellular toxicity, aggregation, and transmission, focusing on a single aspect of its pathology may not be sufficient to halt disease progression. Combining approaches that reduce intracellular α-syn levels, prevent its aggregation, and block its cell-to-cell transmission could provide a more comprehensive strategy for mitigating α-synucleinopathies.

Many biological therapeutics typically cannot penetrate the cell membrane efficiently. As a result, this presents a limitation in treating the intracellular buildup of toxic α-syn aggregates, which is a hallmark of neurodegenerative diseases [185]. Ongoing efforts to develop intracellular targeting strategies, such as nanobodies or cell-penetrating antibody fragments, need to be explored more extensively.

The outlook of targeting membrane receptors and proteins in therapeutic developments is still in the beginning stages. The complexity of receptor functionality limits therapeutic developments to specifically block the pathological interaction between α-syn and these receptors without affecting their normal physiological functions. Although there is substantial potential to develop therapies that can interrupt the spread of α-syn pathology and slow the progression of neurodegenerative diseases, significant challenges remain, including the need for greater specificity in targeting receptors, the development of more effective drug delivery systems, and a better understanding of the underlying mechanisms driving cell-type vulnerability and compensatory pathway.

Intercellular transfer of α-syn through both passive diffusion and active transport mechanisms is another significant pathway for propagating α-syn spread in both neurons and non-neuronal cells. However, the feasibility and efficacy of regulating TNTs and other endocytic or exocytotic pathways present a challenge in developing therapeutics targeting these specific pathways.

Regulating a single pathway to prevent the spread of α-syn might prove to be an inadequate strategy due to the protein’s ability to exploit alternative routes for cell-to-cell transmission. The redundancy and plasticity of cellular mechanisms mean that blocking one avenue could simply result in α-syn finding a different path, thus diminishing the effectiveness of such a targeted approach.

Given the success of immunotherapies in treating various diseases, including cancer and infectious diseases [186], the prospect of employing antibodies as viable treatments for α-synucleinopathies is a promising avenue to consider. In this review, several promising applications of antibodies in addressing α-syn pathology were considered. These include the development of inhibitors targeting LAG3, as well as antibodies that directly interact with α-syn fibrils. While these approaches hold great promise, it is important to acknowledge the numerous considerations and limitations that need to be addressed to advance further in clinical phases. Aside from the key challenge of antibodies crossing the blood-brain barrier, considerations need to be placed on the potential for triggering off-target responses and non-specific inflammatory reactions [187]. 

Other strategic interventions for α-syn pathological agents include gene silencing, which directly halts pathology before monomers aggregate into fibrils and have a chance to propagate. Early administration of siRNA to the substantia nigra may not only slow down the progression of motor symptoms but also have the potential to prevent the neuron-to-neuron transmission of α-syn [136]. Gene silencing may preemptively halt the disease progression before α-syn fibril aggregation, introducing a novel therapeutic opportunity. Nevertheless, a more comprehensive understanding of native α-syn’s role in synaptic plasticity is essential to fully appreciate the implications of this strategy. However, challenges remain in our limited understanding of what roles native α-syn plays in synaptic plasticity. No evidence of reducing SNCA has been shown in humans, thus there is no solid answer to whether this is a realistic therapeutic treatment for α-synucleinopathies.

Overall, the diversity of routes by which α-syn can migrate and amplify within the brain presents a significant obstacle to treatment development. Small quantities of α-syn seeds can induce aggregation, suggesting that therapies might need to target multiple transmission-related pathways simultaneously. While this review has focused on specific pathways and mechanisms related to α-syn cell-to-cell spread and aggregation, it is important to note the existence of other significant targets that are not covered in detail due to categorization reasons. These include accumulation of parkin-interacting substrate due to inactivation of parkin [188, 189], activation of poly(adenosine 5′-diphosphate–ribose) (PAR) polymerase–1 [190], ubiquitination of GCase by the E3 ligase Thyroid Hormone Receptor Interacting Protein 1 [191], and activation of the NLRP3 inflammasome [192]. Each of these targets represents a potential intervention point that could contribute to the development of comprehensive therapeutic strategies for diseases involving α-syn pathology [58]. In addition to the targets discussed, there are several other therapeutic strategies not mentioned in this review that hold promise in tackling α-syn pathology. These include nanozymes [193, 194] and interspecies chimerism for regenerative medicine [195]. When devising therapeutic strategies, it is equally critical to carefully consider and mitigate potential off-target effects. Interfering with membrane receptors and endocytic processes risks disrupting vital cell functions and altering neurotransmitter communication, potentially leading to negative side effects. Indeed, the best forms of therapy need to be specific to pathological agents and their specific transmission pathways, such as neutralizing harmful α-syn forms outside the cell and promoting their uptake and breakdown by glial cells. Despite these challenges, targeting these major steps in α-syn pathology remains a promising therapeutic strategy that warrants further investigation.

**Table 1:** Targeting membrane receptors and proteins

Target	Function	Mechanism	In vivo evidence	In vitro evidence	Intervention	Therapies	
LRP1	Transmembrane receptor that regulates endocytosis of certain ligands	Uptakes and internalize α-syn and mediates spread	Yes	Yes	Inhibition and blocking of α-syn uptake	Limited research	[6–8]
LAG3	Transmembrane protein that regulates immune cell homeostasis	Binding and internalizaion of α-syn PFFs	Yes	Yes	Blocking of α-syn binding	Anti-LAG3 antibodies	[9, 11, 24, 26, 65]
APLP1	Bind proteins on surface of cells	APLP1 and LAG3 co-localize to form complex that binds and internalize α-syn	Yes	Yes	Blocking of α-syn binding	Limited research	[23]
TLR4	Membrane-bound receptor, induces innate immune response	Activation induces autophagy and α-syn clearance but also leads to prolonged inflammation	Yes	Yes	Activation promotes α-syn clearance and blocking reduces neuroinflammation	Limited research	[27–32]
α3-NKA	Membrane-bound protein that maintains electrochemical gradients	α-Syn co-clustering impairs maintenance functions	Prevent clustering and interactions with α-syn	Anti-NKA antibodies	Prevent clustering and interactions with α-syn	Anti-NKA antibodies	[33, 34, 64]
Integrin CD11b	Transmembrane receptor that facilitates cell-matrix adhesion	Binding to α-syn activates NADPH oxidase	Yes	Yes	Blocking α-syn binding attenuates α-syn-induced NOX2 activation	Limited research	[35, 36]
FcγRIIB	Cell surface receptor that mediates immune responses	α-Syn binding inhibits microglial phagocytos and induces α-syn internalization and spread	Yes	Limited research	Blocking of α-syn binding	Limited research	[37–39]
HSPG	Cell surface receptors for proteases and protease inhibitors	Binding and uptake of α-syn PFFs seeds intracellular accumulation	Yes	Yes	Blocking of α-syn binding	Limited research	[25, 44]
PrP^c^	Cell surface glycoprotein and a toxicity-transducing receptor	α-Syn binding triggers phosphorylation and alters calcium homeostasis	Yes	Yes	Blocking of α-syn binding	Limited research	[45–47]
GPNMB	Transmembrane glycoprotein involved with innate immune response	Co-localize and internalize α-syn fibrils	Yes	Yes	Blocking of α-syn binding	Limited research	[48, 52–54]

Specific cell surface receptors and membrane proteins as targets for α-syn-induced pathology and their respective intervention and therapeutic techniques.

**Table 2:** Cell-type specific expression patterns of transmembrane receptors and proteins in the human brain

	Neurons (nPTM)	Astrocytes (nPTM)	Microglial cells (nPTM)	Oligodendrocytes (nPTM)	Oligodendrocyte precursor cells (nPTM)	All cell types (nPTM)
LRP1	37.77 (26.1–55)	107.8	85.9	5.25	162.7	399.42
LAG3	0.16 (0-0.9)	0	0	0	0	0.16
APLP1	53.05 (21.5-120.5)	11.8	11.6	402.45	17.9	496.8
TLR4	0.20 (0-1.3)	48.3	25	0.3	7.1	80.9
α3-NKA	76.62 (17.9-159.9)	8.7	8.1	11.05	13.7	69.28
Integrin CD11b	0.97 (0.5–2.3)	0.6	39.4	0.4	2.8	44.17
FcγRIIB	0	0	0	0.05	0	0.05
HSPG	1.02 (0.3–4.8)	3.4	15.2	3.25	2.4	25.27
PrPc	135.8 (73.1-229.7)	113.9	56.4	133.4	89.1	528.6
GPNMB	0.63 (0-3.6)	0.3	11.6	0.1	52.4	65.03

Cell-type specific expression levels of cell surface receptors and membrane proteins quantified in normalized protein transcript metric (nPTM). Data acquired from The Human Protein Atlas (proteinatlas.org) [56].

**Table 3:** Targeting intercellular spread of ⍺-syn

Target	Mechanism	In vitro evidence	In vivo evidence	Intervention	
Passive diffusion for extracellular release of α-syn	Releases α-syn into extracellular matrix	Yes	Yes	Targeting “naked” α-syn in extracellular matrix	[92, 93, 97]
Exocytosis/vesicular transport	Releases α-syn into extracellular matrix	Yes	Yes	Inhibiting exocytosis and vesicular transport	[98–101]
Tunneling nanotubes	Transports α-syn across extracellular matrix to recipient cells from donor cells	Yes	Yes	Disrupting formation of TNTs	[104–107]
Passive diffusion for cellular uptake of α-syn	Uptakes α-syn into recipient cells	Yes	Yes	Targeting “naked” α-syn in extracellular matrix or mutating sequence motifs on α-syn that modulates binding to plasma membrane	[110–114]
Endocytosis/exosomal uptake	Uptakes α-syn into recipient cells	Yes	Yes	Inhibiting endocytosis and exosomal uptake	[115–117]

Different methods in which α-syn can propagate from cell to cell and their respective intervening strategies.

**Table 4:** Targeting pathological agents

Target	Function	Mechanism	Intervention	
SNCA gene	Encodes for α-syn	Introduces α-syn at the genetic level	Silencing α-syn through RNAi, miRNA, and ASOs	[132, 133, 136, 138–141]
α-Syn oligomers	Neurotoxic protein aggregates of monomers	Induces α-syn pathology and further aggregation into PFFs	Inhibit aggregation and spread through small molecules	[129, 130, 148]
α-Syn fibrils	Aggregated neurotoxic insoluble protein complex	Induces α-syn pathology	Inhibit spreading and internalization through small molecules, antibodies, and nanobodies; active immunization	[131, 146, 147, 149, 154, 156, 163, 165, 167, 170, 171, 173, 174]

Targeting pathological agents as targets for α-syn-induced pathology and their respective intervention techniques

**Table 5:** Gene silencing targeting α-syn in animal models

Method	Target region	Model organism	Outcome	Limitations	
Lentiviral-mediated RNAi	Striatum	Rat	Effective silencing of α-syn	Potential immune response to lentivirus, limited long-term data	[133]
AAV vector-derived RNAi	Substantia nigra	Rat	Long-term knockdown of α-syn without causing neurodegeneration	Possible off-target effects, variability in RNAi efficiency	[135]
AAV vector-derived RNAi	Substantia nigra	Thy1-hSNCA mice and viral hSNCA mice	Reduced α-syn levels and amelioration of behavioral deficits without adverse effects on neurons or glial cells	Limited to specific brain regions, potential off-target effects, long-term efficacy not established	[134]
Polyethylenimine F25-LMW mediated siRNA delivery	Cerebrospinal fluid	Thy1-asyn mice	Reduced SNCA mRNA and protein expression	Concerns about insufficient nucleic acid delivery	[137]
Indatraline-conjugated antisense oligonucleotide	Cerebrospinal fluid	Human WT α-syn overexpression in dopamine neurons mouse	Decreased levels of endogenous α-syn	Select delivery to neuronal populations	[142]
Exosome-mediated delivery of antisense oligonucleotides	Cerebrospinal fluid	α-syn A53T mice	Attenuated α-syn aggregation and decreased degeneration of dopaminergic neurons	Complexity in exosome production and delivery, potential immune response	[143]
Amido-bridged nucleic acids modified antisense oligonucleotide	Cerebrospinal fluid	Human WT α-syn overexpressing PD mice	Downregulated SNCA mRNA and protein, ameliorated neurological defects	Further safety evaluation, controlling for a modest reduction rather than total reduction	[144]
Antisense oligonucleotides	Striatum	Wild-type mice	Local reduction of endogenous α-syn, preventing fibril-induced propagation of pathology	Localized delivery limitations and short duration of observation	[145]

Key studies investigating gene silencing techniques targeting α-syn in various animal models

**Table 6:** Immunotherapy in clinical trials

Name	Sponsor	Target	Mechanism	Status	Result	
**Passive immunizations**						
ABBV-0805	AbbVie/BioArctic	C-terminus	Binds oligomeric/protofibrillar α-syn	Phase 1 completed	Well-tolerable and safe	[156]
				Phase 2 discontinued due to unknown company withdrawn	-	[157, 158]
Lu AF82422	Genmab A/Lundbeck	C-terminus	Binds to all major forms of extracellular α-syn	Phase 1 completed	Well-tolerable and safe	[159]
				Phase 2 completed	Consistent slowing in clinical progression albiet not statistically significant	[160]
UCB7853	UCB	C-terminus	Binds to aggregated α-syn	Phase 1 completed	No results posted	[161]
Cinpanemab (BIIB054)	Biogen/Neurimmune	N-terminus	Binds to aggregated α-syn	Phase 1 completed	Well-tolerable and safe	[162]
				Phase 2 completed	No differences to placebo in progression of motor function, nonmotor function, activities of daily living, quality of life, or imaging biomarkers	[163]
Prasinezumab (PRX002/RG7935)	Roche/Prothena	C-terminus	Binds to aggregated α-syn	Phase 1 completed	Well-tolerable and safe; brain penetration and decreased α-syn levels in free serum	[164]
				Phase 2 completed	No significant slowed decline of PD progression	[165]
				Phase 2b ongoing	-	
**Active Immunizations**						
PD01A	AFFiRiS AG/AC Immune SA	C-terminus	mimics an epitope in C-terminal region of α-syn	Phase 1 completed	Well-tolerable and safe; increased antibodies observed	[167]
				Phase 1 completed	Generated α-syn-reactive IgG in 89% patients	[169]
PD03A	AFFiRiS AG/AC Immune SA	C-terminus	mimics an epitope in C-terminal region of α-syn	Phase 1 completed	Well-tolerable and safe; substantial IgG antibody response	[168]
				Phase 1 completed	Generated α-syn-reactive IgG in 58% patients	[169]
UB-312	United Neuroscience/Vaxxinity	C-terminus	10-amino-acid fragment from C-terminus fused with peptide activating T-helper cells	Phase 1 completed	Well-tolerable and safe; induced antibodies in serum and cerespinal fluid	[170]
				Phase 1b ongoing	-	

Passive and active immunization strategies for targeting α-syn in clinical trials

## Data Availability

Not applicable.

## Abbreviations

α-syn: α-synuclein
PD: Parkinson’s disease
LB: Lewy bodies
DLB: Dementia with Lewy bodies
MSA: Multiple System Atrophy
PAF: Pure autonomic failure
RBD: REM sleep behavior disorder
PFFs: Pre-formed fibrils
LRP1: Lipoprotein receptor-related protein 1
LDL: Low-density lipoprotein receptor
APOE: Apolipoprotein E
Aβ: Amyloid-β
IPSc: Induced pluripotent stem cell
LRP1-ICD: Intracellular domain LRP1
LAG3: Lymphocyte-activation gene 3
mRNA: Messenger RNA
YFP: Yellow fluorescent protein
WT: Wildtype
hep-Tau: Heparin-induced Tau
APLP1: Amyloid precursor-like protein 1
APP: Amyloid precursor protein
TLR2: Toll-like receptor 2
TLR4: Toll-like receptor 4
ROS: Reaction oxygen species
α3-NKA: α3-Na^+^/K^+^-ATPase
NOX2: NADPH oxidase
LC/NE: Locus coeruleus noradrenergic
FcγRIIB: Fc gamma receptor IIB
SHP-1: Src homology 2 domain-containing protein tyrosine phosphatase 1
HSPGs: Heparan sulfate proteoglycans
TAT: Transactivator of transcription
CNS: Central nervous system
PrP^c^: Cellular prion protein
GPNMB: Glycoprotein NMB
DR region: ^897^DVEDSYGQQWTYEQR^911^
AAV: Adeno-associated viruses
ER: Endoplasmic reticulum
TNTs: Tunneling nanotubes
RNAi: RNA interference
shRNA: Short hairpin RNA
miRNA: microRNA
ASOs: Antisense oligonucleotides
SC-D: SynuClean-D
PAR: Poly(adenosine 5′-diphosphate–ribose)
